# Vector autoregression: Useful in rare diseases?—Predicting organ response patterns in a rare case of secondary AA amyloidosis

**DOI:** 10.1371/journal.pone.0289921

**Published:** 2023-08-10

**Authors:** Sandra M. Ihne-Schubert, Malte Kircher, Rudolf A. Werner, Constantin Lapa, Hermann Einsele, Andreas Geier, Torben Schubert

**Affiliations:** 1 Interdisciplinary Amyloidosis Center of Northern Bavaria, University and University Hospital Würzburg, Würzburg, Germany; 2 Department of Internal Medicine II, Hematology, University Hospital Würzburg, Würzburg, Germany; 3 Department of Internal Medicine, Diabetology, Gastroenterology, Tumour Medicine and Palliative Medicine, Medius KLINIK Nürtingen, Nürtingen, Germany; 4 CIRCLE—Centre for Innovation Research, Lund University, Lund, Sweden; 5 Nuclear Medicine, Medical Faculty, University of Augsburg, Augsburg, Germany; 6 Department of Nuclear Medicine, University Hospital Würzburg, Würzburg, Germany; 7 Department of Internal Medicine II, Hepatology, University Hospital Würzburg, Würzburg, Germany; 8 Fraunhofer Institute for Systems and Innovation Research ISI, Karlsruhe, Germany; Kaohsiung Medical University Hospital, TAIWAN

## Abstract

**Background:**

Statistical analyses of clinical data are a cornerstone in understanding pathomechanisms of disorders. In rare disorders, cross-sectional datasets of sufficient size are usually not available. Taking AA amyloidosis as an example of a life-threatening rare disorder resulting from of uncontrolled chronic inflammation, we propose techniques from time series analysis to predict organ response to treatment. The advantage of time-series analysis is that it solely relies on temporal variation and therefore allows analyzing organ response to treatment even when the cross-sectional dimension is small.

**Methods:**

The joint temporal interdependence of inflammatory activity and organ response was modelled multivariately using vector autoregression (VAR) based on a unique 4.5 year spanning data set of routine laboratory, imaging data (e.g., 18F-Florbetaben-PET/CT) and functional investigations of a 68-year-old patient with multi-organ involvement of AA amyloidosis due to ongoing inflammatory activity of a malignant paraganglioma in stable disease for >20 years and excellent response to tocilizumab).

**Results:**

VAR analysis showed that alterations in inflammatory activity forecasted alkaline phosphatase (AP). AP levels, but not inflammatory activity at the previous measurement time point predicted proteinuria.

**Conclusion:**

We demonstrate the feasibility and value of time series analysis for obtaining clinically reliable information when the rarity of a disease prevents conventional prognostic modelling approaches. We illustrate the comparative utility of blood, functional and imaging markers to monitor the development and regression of AA amyloidosis.

## Introduction

Rare diseases are challenging both from a medical and a statistical point of view: Classical study designs and conventional statistical methods are usually not feasible due to limited patient numbers, so that understanding disease evolution, establishment of diagnosis, treatment options, and assessment of treatment response are often based on low evidence. In order to be able to offer these patients adequate diagnostics and treatment, new ways of generating evidence are needed.

A typical example of such a rare disorder represents systemic AA amyloidosis resulting from insufficiently controlled chronic inflammatory syndromes including, e.g. rheumatic diseases, hereditary autoinflammatory diseases, or chronic inflammatory bowel disease [1]. Infrequently, malignancies with inflammatory activity can also induce systemic AA amyloidosis [1]. The therapeutic strategy includes sufficient inflammatory control and avoidance of inflammatory flare-ups, which are known to be associated with poor prognosis [1, 2] by treatment of the underlying malignancy or blocking inflammation e.g. by tocilizumab or anakinra. Up to now, there are no established criteria for assessment of organ involvement and response assessment in AA amyloidosis. Usually, monitoring of sufficient immunosuppression by serum amyloid A (SAA) is used as surrogate as inflammatory control and basis for therapeutic decisions as it represents a predictor of organ response and renal prognosis [1, 3]. The gap of evidence regarding organ response is overcome by transfer of organ response parameters from the more common form light chain (AL) amyloidosis. Therefore, proteinuria is used for assessment of renal response and alkaline phosphatase (AP) as surrogate for hepatic involvement. Cardiac involvement is rare in AA amyloidosis (about 10%) and does not show the typical echocardiographic phenotype as known from AL and transthyretin (ATTR) amyloidosis. However, cardiac biomarkers such as N-terminal pro-B-natriuretic peptide (NT-proBNP) and troponins are used for cardiac response assessment in analogy to AL and ATTR amyloidosis. Because pathomechanisms differ substantially between AL and AA amyloidosis, the application of AL amyloidosis related biomarkers may be misleading.

Moreover, organ response patterns and time to organ response differ within and between the subtypes. The rarity and inhomogeneity of this entity makes it difficult to generate evidence, especially as there is little statistically reliable information on organ response patterns from larger patient collectives from the rare subtypes. Statistical attempts to model interdependence of inflammatory activity and organ response has not been published up to now to our knowledge.

Using one of seven worldwide known cases of AA amyloidosis in inflammatory active paraganglioma [4] as prototypical example, we here provide an in-depth characterization of the development and regression of AA amyloidosis manifestations by both established and innovative methods. In specific, we demonstrate the value of time series modelling by vector autoregression (VAR) as an innovative approach to characterize the mutual interdependence of inflammatory markers and ensuing organ response [5]. VAR, and techniques from time-series analysis in general, have been widely applied in a many disciplines such as economics, bio-statistics or physics and their statistical properties are well understood. While only few applications in medicine exist so far [6, 7], the key advantage of VAR modelling is that the identification of organ response is possible even when data of only one “case” is available, which is observed frequently over time. The time series identification makes VAR particularly interesting in rare diseases such as AA amyloidosis because here patients are few but are usually under detailed clinical surveillance over time.

## Material & methods

The clinical development of amyloidosis in the case presented was retrospectively analyzed using the available medical documentation from 1990 until 2016 of the attending general practitioner and specialists as well as continuous oncologic staging results documented in-house since 1996. Extensive medical history and clinical evaluation regarding amyloidosis manifestations at onset and under treatment were prospectively monitored in an uninterrupted manner every three to four months from 2016 onwards. Laboratory evaluations followed the standard for AA amyloidosis patients at the local Interdisciplinary Amyloidosis Center of Northern Bavaria since November 2017. The analysis was performed in the framework of the local amyloidosis cohort study AmyKoS covered by ethic vote 48/18 of the Ethics committee of the University Würzburg. The patient provided written consent regarding analysis and publication of her clinical data.

Definition of organ involvement in amyloidosis was performed according to criteria published by Gertz et al. 2005 [8], updated in Gertz et al. 2010 [9]. Cardiac work-up included the measurement of the cardiac biomarkers NT-proBNP and high-sensitive troponin T (hsTnT), electrocardiogram, transthoracic echocardiography, and ^18^F-Florbetaben-PET/CT. Renal assessment was based on estimated glomerular filtration rate (eGFR) as well as creatinine clearance, proteinuria and albuminuria measured from 24-hour urine samples. Hepatic involvement and function were tracked by alkaline phosphatase (AP), bilirubin, gamma-GT, transaminases (ALT, AST), as well as dynamic liver function measured by ^13^C-methacetin breath test. Liver transient elastography was used as new tool according to Richards et al. [10].

Staging of malignant paraganglioma included thoracic computed tomography (CT) scan, magnet resonance imaging (MRI) of liver and spine twice per year, and bone scintigraphy once per year, according to the local standard of care.

Prospectively assessed transthoracic echocardiograms were done by internally certified sonographers of the Academic Core Lab Ultrasound-based Cardiovascular Imaging using a high-definition ultrasound machine Vivid E95, GE, with the same system presets during the prospective evaluation. Stored images were analyzed off-line using EchoPAC (General Electrics). Retrospectively analyzed echocardiograms followed local standard of care. Imaging by whole-body planar scintigraphy and ^18^F-florbetaben-PET/CT and imaging analysis were performed as previously described by Kircher et al. 2019 [11]. Injected activity was 470 to 636 MBq (mean 576 ± 75.5 MBq) for ^99m^Tc-DPD whole-body planar scintigraphy and 293 to 318 MBq (mean 299 ± 14.1 MBq) for ^18^F-florbetaben-PET/CT. For estimation of amyloid burden, percentage of myocardial tracer retention (retention index, RI, given in [%]) was calculated as the change in left ventricular myocardial standardized uptake value (SUV_mean_) on summed-framed images of the first 5 min after i.v. injection of the tracer (SUV_0–5_) and between 15 and 20 min (SUV_15–20_), based on the formula used and published by Law et al. 2017 [12] and Kircher et al. 2019 [11]: RI = [1−(SUV_0−5_–− SUV_15−20_ SUV_0−5_)]∙100%. Quantification of PET data was performed by the software PMOD, version 3.7 (PMOD Technologies LLC, Zuerich, Switzerland). Liver transient elastography was performed on a Fibroscan® 502 touch with software version 1.40 according to the manufacturer’s instructions.

## Data analysis

Statistical analysis was performed using STATA® version 14. Pairwise non-parametric correlation analysis of pooled information of biomarkers and imaging markers was performed (Spearman). To illustrate the joint temporal interdependence of inflammatory activity and organ response, we multivariately modelled time series of iteratively measured markers using vector autoregression (VAR) [5]. In VAR, each parameter $X_{t}=(x_{1t},\ldots ,x_{kt})$ is described as a function of its own past value and the past values of all other parameters (and an additional vector of contemporaneous variables Z_t_ including at least the regression constant). VAR models can account for higher order lag periods, i.e. the number of time periods between a past measurement value (t-1) and a current value (t). Applying the information criteria of Akaike as well as Bayes and Schwarz, we found that the 1-lag period yielded the best model fit. Due to decreasing frequency of the data points and to avoid extended data imputation, only values up to day 1438 (n = 208 observations) were considered in the VAR analysis so that the mean period length, i.e. 1 lag, was approximately 6 days. In vector notation, a VAR(1)– 1, which indicates the number of lags, can be expressed as follows:

$$X_{t}=BX_{t-1}+AZ_{t}+u_{t}$$ (1)

where u_t_ is an error-term. A and B are matrices of coefficients to be estimated, with A determining in particular, how the clinical parameters depend on each other others as well as their own past developments. Explaining clinical parameters by their own past is a reasonable approach in contexts where it can be assumed that past developments have prediction power for future developments. In all types of amyloidosis this is very likely to be the case because the relevant clinical parameters are used as expression of organ damage. Since the organ damage does not immediately revert, it accumulates over time, which creates a stable relationship between the clinical parameters and their past developments. VAR-models precisely capture this time dependence and allow analyzing how the parameters depend on each other over time. Notably, we can analyze whether a lagged value of one parameter helps predicting another one in the future. We can also estimate how much time this response takes to unfold. And finally, it allows analysing how quickly a parameter returns to its normal level after experiencing a shock (for example after treatment or a worsening of the condition). In the next subsection, we will provide evidence on these questions in our particular case of AA amyloidosis.

Technically, estimating VAR-models relies on complete data and thus requires the imputation of missing values. We used a Monte-Carlo based simulation model for multiple imputation, where missing values are drawn based on chained regression analysis using truncated normal regression to ensure that imputed values are positive [13, 14].

To visualize, how a change in one marker may affect the level of other markers at later time points, we calculated impulse-response functions (IRF). By artificially altering the error-term of a marker at a certain time point by one standard deviation (“impulse”), one thus can monitor, how strong and for how long this impulse may affect the other variables (“response”).

## Results

### Summarised case study with in-depth details

**Fig 1:**
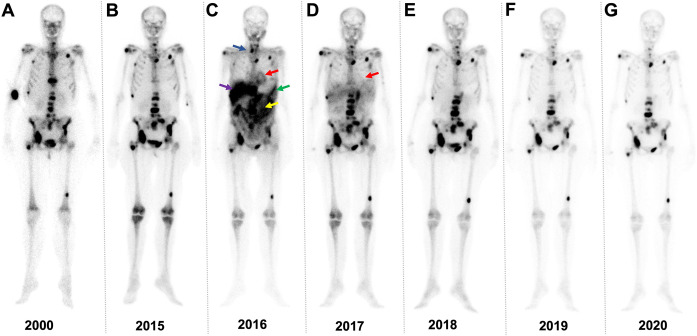
Tracking amyloid by whole body bone scintigraphy. Time series of bone scintigraphy imaging with ^99m^Tc 3,3-diphosphono-1,2-propanodicarboxylic acid (DPD). (A) In 2000, multiple osseous sites of disease attributable to paraganglioma can be appreciated throughout the skeleton. Patient remained stable for another 15 years (B). In 2016, cardiac (red), intestinal (yellow), hepatic (purple), splenic (green) and thyroid (blue) accumulation of the bone-seeking radiotracer can be appreciated, supporting the notion of onset of amyloidosis (C). After initiation of anti-amyloid treatment, a significant reduction of DPD in cardiac (red arrow in D) and extra-cardiac manifestations was noted. Both amyloid manifestations and metastatic lesions in the skeleton remained stable in (E) 2018, (F) 2019 and (G) 2020.

The clinical aspects of the analysed case of inflammatory active paraganglioma with associated AA amyloidosis has already been published elsewhere [4]. For a better understanding, we briefly summarize the case study [4] and include additional in-depth details on organ response pattern: A glomus tumour close to the bifurcation of the carotid artery was surgically removed in 1990. Seven years later, metastases were detected for the first time. Since then, ongoing treatment with octreotide resulted in stable disease. An acute phase reaction since at least 1995 was finally interpreted as paraganglioma-associated, but octreotide showed no effect on tumour associated inflammation. As long-term complication of the ongoing inflammation the patient developed rapid progressive systemic AA amyloidosis with multi-organ involvement including cardiac and renal, hepatic, gastrointestinal, spleen and thyroid gland involvement within less than one year according (Fig 1).

**Fig 2:**
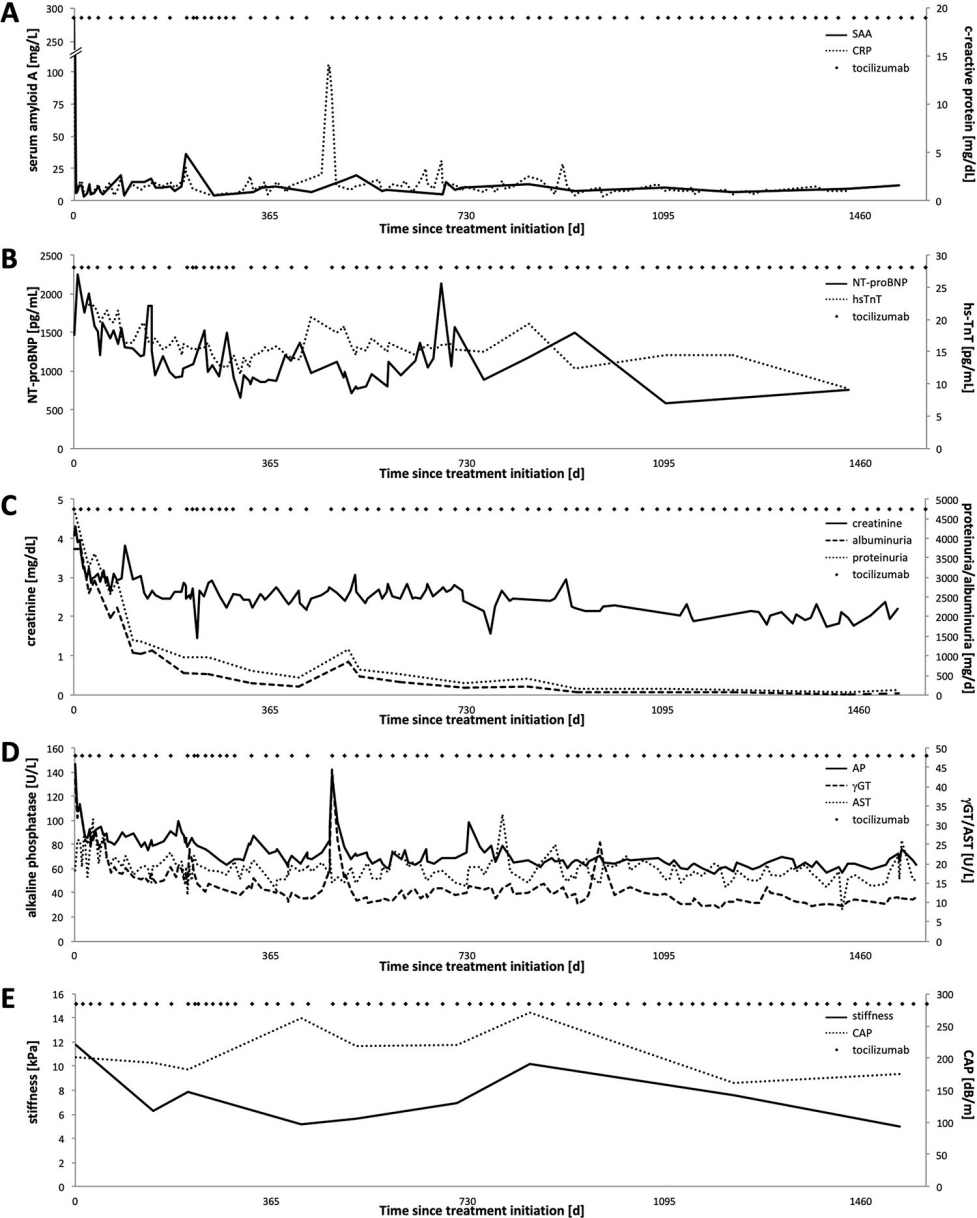
Inflammatory activity and organ function/response under treatment with tocilizumab. Graphical time series of markers of inflammatory activity (A) and conventional and innovative cardiac (B), renal (C) and hepatic organ function and response (D, E). The application time points of tocilizumab are shown as black diamonds. (A) Inflammatory activity. Immediately after initiation of anti-inflammatory therapy, there is a suppression of SAA levels at <10 mg/L (solid black line). Short-term increases correlate with prolonged tozilizumab application intervals. C-reactive protein behaves analogously (dotted line). (B) Cardiac response. NT-proBNP (solid black line) shows a long-term declining trend with short-term fluctuations that are not accompanied by parallel changes in inflammatory activity or are temporally not related to changes in tocilizumab application intervals. High-sensitive troponin T (dotted line) behaves analogously over time. (C) Renal response. Creatinine shows a slow but steady improvement with persistent renal insufficiency (solid black line). Proteinuria (dotted line) and albuminuria (dashed line) normalize long-term. (D) Hepatic response. AP normalizes within 14 days. Increasing inflammatory activity is immediately reflected in analogous short-term fluctuations in AP. GGT (dashed line) runs parallel to AP. AST as example for transaminases is shown as dotted line with stable values long-term. (E) Hepatic response. Parallel to the regression of the scintigraphically detectable manifestation, there is a decrease in liver stiffness (solid black line) with a one-time, not clearly explainable increase on day 846. CAP values are stable in the long-term course (dotted line).

**Fig 3:**
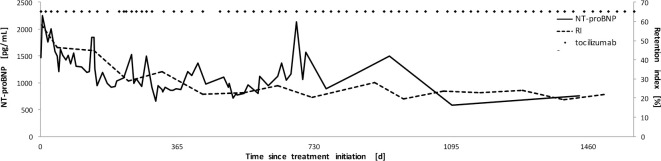
Tracking cardiac response by serial ^18^F-Florbetaben-PET/CT—Times series of retention indices measured by serial ^18^F-Florbetaben-PET/CT in comparison to cardiac biomarker NT-proBNP. NT-proBNP values (black line) decrease continuously over time with short-term fluctuations. Retention indexes (dotted line) map the long-term course in a similar way. The time points of tocilizumab application are noted by means of black dots.

Off-label treatment with tocilizumab resulted in immediate and sufficient suppression of inflammation using SAA and C-reactive protein (CRP) as surrogate parameters for inflammation (Fig 2A). Short phases of increased inflammatory activity were observed in the temporal context of dose reduction of tocilizumab due to haematotoxicity. Organ response was continuously evaluated and multi-modally monitored over the course of the following 4.5 years (Fig 2). The initially suspected cardiac involvement showed a continuous decrease of the retention index (RI) measured by ^18^F-Florbetaben-PET/CT, which normalized within 14 months of treatment (from 58.0% to 22.1%) (Ihne et al. 2022 [4]; Fig 3). Cardiac uptake in bone scintigraphy regressed until 2018 and has remained negative ever since (Fig 1). As echocardiography at presentation was not indicative of cardiac amyloidosis, it was regarded inconclusive for cardiac monitoring [4]. A decrease in cardiac biomarkers NT-proBNP and hsTnT, however, was observed (Fig 2B). Intermittent increases of cardiac biomarkers, especially NT-proBNP, plausibly reflected the short phases of increased inflammatory activity due to dose reduction of tocilizumab mentioned above. Correspondingly, renal function improved continuously, with increase in GFR of 19 mL/min to 33 mL/min and decrease in proteinuria and albuminuria from 4.7 g/day and 3.7 g/day to a minimum of 0.08 g/day and 0.021 g/day (Fig 2C), respectively. Liver involvement also responded well, with initial AP levels of 147 U/L normalizing within 14 days of treatment (Fig 2D). The initially increased stiffness values of 11.8 kPa in the fibroscan showed a decrease to 5 kPa (Fig 2E). Organ involvement of liver, spleen, gastrointestinal tract, and thyroid gland as documented by bone scintigraphy regressed completely and remained negative from 2019 onwards (Fig 1). Latent hypothyroidism receded and hormone substitution was reduced.

### Predicting organ response by time series analysis of biomarkers

The wealth of laboratory and functional data collected from September 2016 to December 2020 resulted in 208 data points across a period of 53 months (Fig 2; Table 1): inflammatory activity (SAA, n = 45; CRP, n = 122; blood sedimentation rate (BSR), n = 84), cardiac involvement (NT-proBNP, n = 69; hsTnT, n = 67; RI, n = 16), renal involvement (eGFR, n = 183; proteinuria, n = 22; albuminuria, n = 23; creatinine clearance in 24-hour urine, n = 16), and hepatic organ involvement (AP, n = 160; γGT, n = 167; AST, n = 167; liver stiffness, n = 9). Pooled pairwise correlation analysis (Table 1) demonstrated the expected high degree of correlation amongst inflammatory markers (e.g., for CRP and SAA: rho 0.71), but also significant correlation between CRP and AP (rho 0.39) as well as RI (rho 0.81). By contrast, CRP was not significantly related with cardiac markers. RI was closely related to AP (rho 0.92), but did not reach significance regarding NT-proBNP and hsTnT (rho 0.71 and 0.60, respectively). Correlation of inflammatory markers and AP with liver stiffness also did not reach significance. The correlation of inflammatory activity with proteinuria and albuminuria was not significant. However, AP was strongly correlated with proteinuria and albuminuria (rho 0.96 and 0.94, respectively), but also to a smaller degree with cardiac biomarkers (rho 0.37 and 0.57, respectively).

**Table 1:** Correlation matrix of laboratory and functional markers of inflammatory activity and organ function.

Marker		CRP	SAA	BSR 1h	BSR 2h	NT-proBNP	hs-TNT	Creatinine	eGFR	Proteinuria	Albuminuria	Creatinine clearance	AP	Bilirubin	γGT	AST	Liver stiffness
**SAA**	rho	0.71															
	p	<0.001															
	n	39															
**BSR 1h**	rho	0.55	0.32														
	p	<0.001	0.064														
	n	62	34														
**BSR 2h**	rho	0.56	0.35	0.98													
	p	<0.001	0.042	<0.001													
	n	62	34	84													
**NT-proBNP**	rho	0.04	-0.13	-0.14	-0.15												
	p	0.794	0.501	0.303	0.255												
	n	53	29	56	56												
**hsTnT**	rho	0.17	0.16	0.12	0.15	0.48											
	p	0.244	0.413	0.368	0.261	<0.001											
	n	51	27	55	55	66											
**Creatinine**	rho	0.20	-0.14	0.08	0.11	0.43	0.63										
	p	0.043	0.394	0.496	0.365	<0.001	<0.001										
	n	101	39	74	74	65	62										
**eGFR**	rho	-0.27	0.12	-0.11	-0.14	-0.39	-0.58	-0.96									
	p	0.003	0.431	0.334	0.199	<0.001	<0.001	<0.001									
	n	122	43	83	83	69	67	139									
**Proteinuria**	rho	0.45	0.34	0.31	0.39	0.92	0.87	0.88	-0.92								
	p	0.095	0.344	0.347	0.235	<0.001	<0.001	<0.001	<0.001								
	n	15	10	11	11	12	12	15	19								
**Albuminuria**	rho	0.40	0.34	0.31	0.39	0.93	0.81	0.84	-0.90	1.00							
	p	0.125	0.344	0.347	0.235	<0.001	<0.001	<0.001	<0.001	<0.001							
	n	16	10	11	11	13	13	16	20	22							
**Creatinine clearance**	rho	-0.35	-0.19	-0.16	-0.25	-0.92	-0.88	-0.91	0.91	-0.82	-0.85						
	p	0.237	0.651	0.713	0.548	<0.001	<0.001	<0.001	<0.001	<0.001	<0.001						
	n	13	8	8	8	10	10	12	16	15	16						
**AP**	rho	0.39	-0.03	0.15	0.15	0.37	0.57	0.68	-0.70	0.96	0.94	-0.91					
	p	<0.001	0.872	0.205	0.198	0.003	<0.001	<0.001	<0.001	<0.001	<0.001	<0.001					
	n	108	39	74	74	65	63	124	160	16	17	13					
**Bilirubin**	rho	-0.09	-0.11	-0.49	-0.50	0.03	-0.36	-0.01	0.05	-0.31	-0.33	0.05	0.09				
	p	0.407	0.543	<0.001	<0.001	0.867	0.013	0.911	0.551	0.274	0.252	0.881	0.285				
	n	91	31	50	50	47	46	102	139	14	14	11	131				
**γGT**	rho	0.21	-0.17	0.08	0.06	0.51	0.59	0.67	-0.67	0.98	0.98	-0.90	0.83	0.06			
	p	0.024	0.284	0.488	0.618	<0.001	<0.001	<0.001	<0.001	<0.001	<0.001	<0.001	<0.001	0.497			
	n	112	40	75	75	65	63	128	167	17	18	14	159	167			
**AST**	rho	-0.09	-0.51	-0.24	-0.25	0.36	0.30	0.34	-0.29	0.60	0.60	-0.53	0.42	0.18	0.41		
	p	0.348	<0.001	0.038	0.029	0.003	0.016	<0.001	<0.001	0.011	0.008	0.051	<0.001	0.034	<0.001		
	n	111	39	75	75	65	63	128	167	17	18	14	160	166	167		
**Liver stiffness**	rho	0.71	0.60	0.70	0.60	0.10	0.60	0.70	-0.49	1.00	0.70	-0.20	0.45	-0.74	0.55	-0.26	
	p	0.111	0.285	0.125	0.208	0.873	0.285	0.188	0.217	<0.001	0.188	0.747	0.260	0.092	0.160	0.528	
	n	6	5	6	6	5	5	5	8	4	5	5	8	8	8	9	
**Retention index (RI)**	rho	0.81	-0.11	0.41	0.37	0.71	0.60	0.87	-073	NA	NA	NA	0.92	-0.44	0.99	0.36	NA
	p	0.027	0.894	0.355	0.403	0.111	0.284	<0.001	<0.001	NA	NA	NA	<0.001	0.271	<0.001	0.332	NA
	n	7	4	7	7	6	5	11	11	NA	NA	NA	9	8	9	9	NA

Data are Spearman correlation coefficients (rho) of pairwise comparisons; n = number of measurements

CRP = C-reactive protein; SAA = serum amyloid A; BSR 1h / 2h = blood sedimentation rate after 1 / 2 hours; NT-proBNP = N-terminal pro-B-type natriuretic peptide; hsTnT = high-sensitive troponin, eGFR = estimated glomerular filtration rate according to MDRD formula; creatinine clearance = measured glomerular filtration rate from 24-hour urine sample; AP = alkaline phosphatase; γGT = γ-glutamyltransferase; AST = glutamate oxaloacetate transaminase; liver stiffness = liver stiffness measured by fibroscan®; N/A = not available

Retention index was measured by ^18^F-florbetaben-PET/CT.

Since correlation analysis does not allow insights into the temporal dependence of investigated variables, a VAR analysis was performed. VAR allowed modelling the interactions between inflammatory activity and organ improvement over time, in particular for variables already validated for AL amyloidosis or those correlated with indicators of inflammation. We chose CRP for inflammatory activity, and NT-proBNP, AP, and proteinuria for cardiac, liver, and renal involvement/function. We preferred CRP over SAA, because it was recorded substantially more often and thus required less imputation. RI and liver stiffness could not be analyzed using this model because of too few data points. Table 2 shows that, e.g., AP measured at a certain time point, i.e. AP (t), was positively affected by the CRP value measured one lag period ahead, i.e. CRP (t-1) (b = 1.40, p<0.05). Neither NT-proBNP nor proteinuria were affected by CRP in a similar manner. Past AP weakly impacted on proteinuria (b = 31.76, p<0.1, Column 3), while no effect emerged into the opposite direction (b = 0.0002, p>0.1).

**Table 2:** Multivariable time series analysis for inflammatory and organ response markers with one lag period.

	AP (t)	NT-proBNP (t)	proteinuria (t)	CRP (t)
AP (t-1)	0.46***	6.48	31.76*	-0.02
	(2.72)	(1.30)	(1.77)	(-1.23)
CRP (t-1)	1.40**	-8.55	-9.42	0.70***
	(2.18)	(-0.30)	(-0.13)	(11.51)
NT-proBNP (t-1)	0.00	0.06	0.07	-0.00
	(0.18)	(0.40)	(0.13)	(-0.12)
Proteinuria (t-1)	0.00	0.01	0.11	0.00
	(0.10)	(0.11)	(0.47)	(0.07)

Columns 1–4 represent the four regressions of our vector autoregression (VAR) informed by n = 208 observations across 1400 days. One lag period indicates about 6 days. Coefficients are presented with t-values in parentheses providing an estimate of the comparative strength of the association.

* p<0.10

** p<0.05

*** p<0.01

Interpretation guide: Column 1 expresses AP(t) as a function of AP(t-1), CRP(t-1), NT-proBNP(t-1) and proteinuria(t-1). For example, the coefficient on CRP(t-1) of 1.40 means that a one-unit increase of CRP(t-1) increases AP(t) by 1.40 units one lag period later.

**Fig 4:**
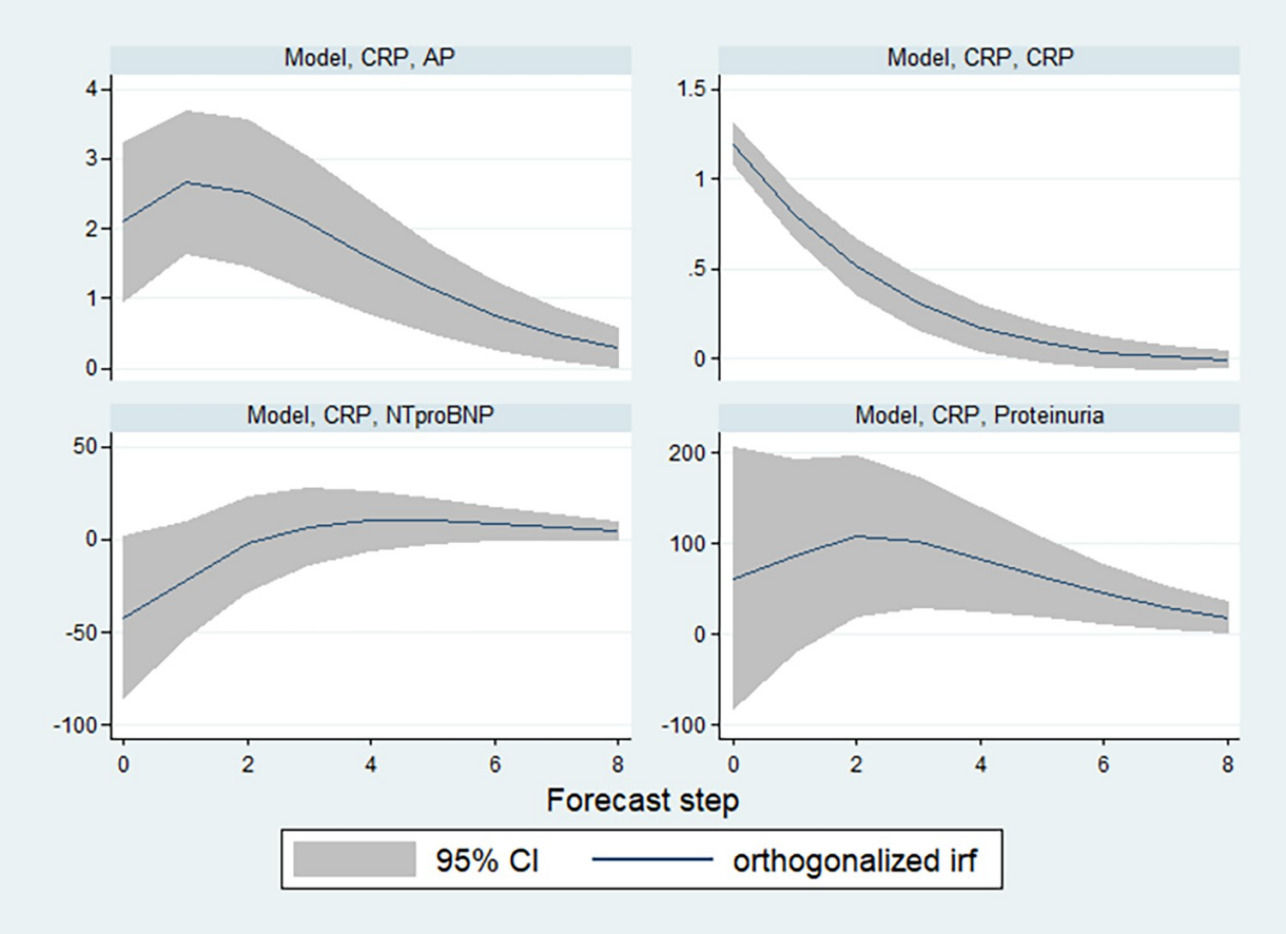
Impulse response functions (IRF) plotting the evolution of the model parameters after a simulated increase in CRP. The IRF models the exogenous increase in CRP induced by a one-standard-deviation impulse to the error-term in the CRP regression. Upper left panel shows the effects on AP: A significant and initially accumulating increase in periods 0 and 1 is apparent, which slowly returns to normal after 8 periods (48 days). Upper left panel shows the effects on CRP: Observable is an instantaneous significant increase in period 0, which returns to normal after 5 periods (30 days). Lower left panel shows the effects on NT-proBNP: There are no statistically significant effects. Lower right panel shows the effects on proteinuria: Observable is a significant and initially accumulating increase in periods 0, 1 and 2 which slowly returns to normal after 8 periods (48 days).

Impulse-response functions (IRF) were calculated to visualize, how an increase in CRP may affect levels of NT-proBNP, AP, and proteinuria. Fig 4 exemplifies IRF plots for CRP with 95% confidence intervals (CI). The lower right panel shows that after the CRP impulse returns to normal after about 5 forecast steps (i.e., 5 lag periods = 5 x 6 days). Whereas a CRP impulse had no impact on NT-proBNP, as the 95% CI intervals always included zero, there were significant increases both for AP and proteinuria. AP reacted with an instantaneous jump to the new level from which it started returning to the former level after 1 lag period (i.e., after 6 days). It remained significantly elevated until lag period 8 (48 days). By contrast, proteinuria instead started to accumulate from its old level and reached a peak after 2 periods (12 days) and then fell back to the pre-impulse levels. Its level was significantly elevated between period 1 (day 6) and period 8 (day 48). Overall, the response in AP appeared to be stronger and faster as compared to proteinuria.

As VAR investigates a flexible system, we performed various sensitivity analyses that all confirmed the robustness of the above highlighted findings: a) replacing CRP by SAA; b) applying different methods of data imputation; c) including an additional explanatory variable describing the time-lapse between consecutive measurements that were not equidistant as well as the exact dates of tocilizumab application; d) restricting the sample period to the first 1000 days, where clinical measurements were more frequent e) re-running models taking into account second or third order partial autocorrelations; f) implementing longer lag periods (i.e., 1, 3, 6, 9, or 12 months); g) adding eGFR and creatinine to model effects of renal impairment.

## Discussion

The association of AA amyloidosis with solid tumors as well as hematological neoplasms is rare, but has been described in case reports[15, 16]. The case presented here is unique in terms of its detailed multi-marker work-up over a period of several years allowing to analyze the development and response of AA amyloidosis in a multimodal fashion.

Our patient responded extremely well to tocilizumab, which is consistent with its efficacy in AA amyloidosis as reported previously in several case reports and small case series. Tocilizumab was applied intravenously every 2–4 weeks at doses of 8 mg/kg body weight. According to Lane et al., a significant reduction in SAA could be achieved already within 10 days after the first application (70 mg/L initially, 4 mg/L after 10 days) [17]. Under continued tocilizumab treatment, a regression up to the complete disappearance of the gastrointestinal amyloid deposits has been observed earlier [17, 18]. Similarly, favorable renal response as indicated by a significant reduction in proteinuria up to complete normalization has been reported [18, 19], as well as a reduction in serum creatinine, especially in severe cases (patient 8 in the case study of Yilmaz et al. [19]). Response rates were 33% improvement and 33% stable disease in a multi-center study following 9 non-dialysis-dependent AA amyloidosis patients with renal involvement [20]. Consistently, Miyagawa et al. observed an improvement in renal function including proteinuria in 4 out of 5 patients [21]. Tocilizumab also appears to be an effective therapy in nephrotic syndrome and rapidly progressive AA amyloidosis [22]. Cardiac involvement in AA amyloidosis is rare and indicates a particularly poor prognosis. In cases reported by Matsui et al. and Hattori et al., treatment with tocilizumab markedly improved cardiac amyloidosis [23, 24].

As described above, there are so far no established criteria for assessment of organ involvement and response assessment in AA amyloidosis beyond monitoring of adequate suppression of inflammatory activity by SAA. In clinical routine, this gap is bridged by transferring the parameters used in other subtypes of amyloidosis. Current definitions for organ involvement and response are primarily limited to light chain (AL) amyloidosis [25, 26] and employ unspecific parameters that are frequently affected by comorbid conditions such as renal impairment as demonstrated in this case of AA amyloidosis. Therefore, the non-invasive diagnosis of cardiac involvement in patients with extra-cardiac histopathological evidence of AL amyloid is based on “characteristic morphological findings” and elevation of cardiac biomarkers as NT-proBNP or troponin [8, 9, 27, 28]. Morphological findings include increased chamber wall thickness and apical sparing, which occurs only late during disease progression. NT-proBNP is a sensitive marker facilitating the monitoring of cardiotoxicity of the free light chains and response to treatment [29–31], but strongly dependent on determinants as fluid retention, renal function, and medication (e.g. immunomodulators [32]). Several staging systems have been published, summarized in Ref. [27], allowing to estimate the mortality risk [27, 33–37]. Renal involvement is defined by proteinuria. The risk of dialysis can be estimated using total proteinuria and renal function according to the renal staging system of Palladini et al. [38]. AP and hepatomegaly are established markers for hepatic involvement. Usually, treatment decision in AL amyloidosis is based on the hematological response after 3 and 6 months of treatment, which is a strong prognostic predictor for organ response. Importantly, markers of early organ response are lacking as available markers for direct measurement of organ response react with delay [26, 30]. A grading and estimation of the delay of organ response in AL amyloidosis was proposed by Muchtar et al. [39]. In ATTR amyloidosis, few staging systems addressing the degree of cardiac involvement have been published [40, 41]. Response criteria are not established yet due to lacking treatment options in the past, but are now urgently required in view of their increasing availability [42]. Furthermore, there is a clear need of more specific and robust markers that ideally can be applied across amyloidosis subtypes.

A VAR modelling approach was used in amyloidosis for the first time and might enable to overcome the described difficulties: The current case report prototypically details the multi-organ involvement and organ response using conventional markers established to monitor disease progression in amyloidosis, e.g. AP, proteinuria and NT-proBNP, and dynamically analyzes their association with underlying inflammatory state.

Expectedly, direct dependence of AP as a fast reacting parameter (normalization within 14 days after initiation of treatment) on inflammatory activity was apparent. A concomitant decrease in NT-proBNP was not directly correlated with inflammatory activity, which might be explained partially by the sensitivity of NT-proBNP to confounding factors, in particular the dynamics of renal insufficiency and fluid retention. Further, rapid decreasing NT-proBNP levels indicative of cardiac response in AL amyloidosis are attributed to reduction of direct cardiotoxicity by reduced levels of circulating free light chains under treatment. However, AL amyloid deposits remain in the tissue and regress over time only slowly. In AA amyloidosis, there is no known direct toxicity of the precursor protein so that an immediate reaction of cardiac biomarkers should not be expected. Consistently, imaging tools such as MRI measuring extracellular volume react with a delay of several months. In support of this reasoning, we observed a correlation of NT-proBNP, inflammatory activity, and RI in our patient. The latter is thought to reflect amyloid burden in the heart.

The result that AP levels, but not inflammatory activity at the previous measurement time point predicted proteinuria may be explained by the greater inertia of proteinuria compared to AP. Thus, response in one organ might predict organ response in the other organ as expected in analogy to AL amyloidosis.

The here presented data exemplify a single case and any hypotheses derived from such data require confirmation in larger cross-sectional samples to rule out case-specificities. As real-world data include gaps resulting from clinical needs of the patient and limitations such as radiation exposure, feasibility in routine practice, appropriateness of diagnostics burdening the patient and generating costs, imputation of missing values was required to enrich the VAR model. However, contemporary methods and robustness checks were used to strengthen the validity of the VAR modelling approach.

## Conclusion

We multi-modally tracked the development of AA amyloidosis and organ response to tocilizumab over a time course of 4.5 years. Time series analyses of correlations between inflammatory activity and parameters of organ function and treatment response allowed to bridge the lack of cross-sectional data and the resulting failure of conventional statistical methods. They confirmed known patterns from other subtypes, but underlined limitations of markers such as NT-proBNP when used in this context.
